# Draft Genome Sequence of a Novel Species of *Halococcus* (Strain IIIV-5B), an Endophytic Archaeon Isolated from the Leaf Tissue of Avicennia germinans

**DOI:** 10.1128/MRA.00421-20

**Published:** 2020-05-21

**Authors:** Jeysika Zayas-Rivera, Yadiel Rivera-Lopez, Madeline Velázquez-Méndez, Nicole Romero-Oliveras, Rafael Montalvo-Rodríguez

**Affiliations:** a Biology Department, University of Puerto Rico, Mayagüez, Puerto Rico; b Department of Plant Pathology, University of Wisconsin—Madison, Madison, Wisconsin, USA; Portland State University

## Abstract

Strain IIIV-5B was isolated from the leaf tissue of the black mangrove, Avicennia germinans. This microorganism belongs to the genus *Halococcus*. Here, we present the draft genome sequence of strain IIIV-5B, a novel species of this genus. The 3,869,808-bp genome has a G+C content of 63.9% and around 3,812 coding sequences.

## ANNOUNCEMENT

Mangroves are woody plants often located in the intertidal areas of tropical and subtropical regions (1) that provide a suitable habitat for multiple species and contribute to the stabilization of coastlines by preventing erosion and protecting the shore from tidal waves (2–8). A novel strain (IIIV-5B) belonging to the genus *Halococcus*, first reported by Schoop in 1935 (9), was isolated and characterized during a prokaryotic biodiversity survey of halophilic and halotolerant endophytes in leaves of Avicennia germinans at the solar salterns in Cabo Rojo, Puerto Rico. Here, we present the draft genome sequence of this putative novel species, as reports of endophytic haloarchaea are scarce.

Strain IIIV-5B was isolated from surface-sterilized leaf tissue in Sehgal-Gibbons medium (10) containing 15% NaCl (wt/vol). The leaf tissue was placed onto the surface of solid medium and incubated at 30°C. Growing colonies were selected and purified by plate streaking until pure cultures were obtained. Genomic DNA extraction was performed using the Promega Wizard genomic DNA purification kit and sequenced at MicrobesNG in Birmingham, United Kingdom. Genomic DNA libraries were generated using a Nextera XT library prep kit (Illumina, San Diego, CA, USA) following the manufacturer’s instructions, with the exception of the use of 2 ng of DNA instead of 1 ng as input and an increase of the PCR elongation time to 1 min. Pooled libraries were quantified using the Kapa Biosystems library quantification kit for Illumina. Libraries were sequenced using an Illumina HiSeq instrument (250-bp paired-end protocol). Adapters were trimmed with Trimmomatic 0.30 (11), *de novo* assembly was performed using SPAdes version 3.7 (12), and contigs were annotated with Prokka 1.11 (13). Genome sequences were then analyzed using the Rapid Annotations using Subsystems Technology (RAST) server (14–16). A draft genome sequence of 3,869,808 bp was assembled using 74 contigs (Table 1). Features in this genome include a G+C content of 63.96%, 2 CRISPR repeats, and around 3,812 coding sequences, 3 of which are suggested to encode rRNAs and 49 of which are suggested to encode tRNAs. Similar values were obtained with the NCBI Prokaryotic Genome Annotation Pipeline (3,877,752 bp, 63.9% G+C content, and 3,812 coding sequences).

**TABLE 1:** Genome statistics for strain IIIV-5B

Statistic	Value
Sequencing	
No. of reads	279,550
Genome coverage (×)	30
Assembly	
Size (bp)	3,869,808
No. of contigs	74
G+C content (%)	63.96
*N*_50_ (bp)	99,920
Gene models	
Total no. of coding sequences	3,812
Total no. of RNAs	49

**FIG 1:**
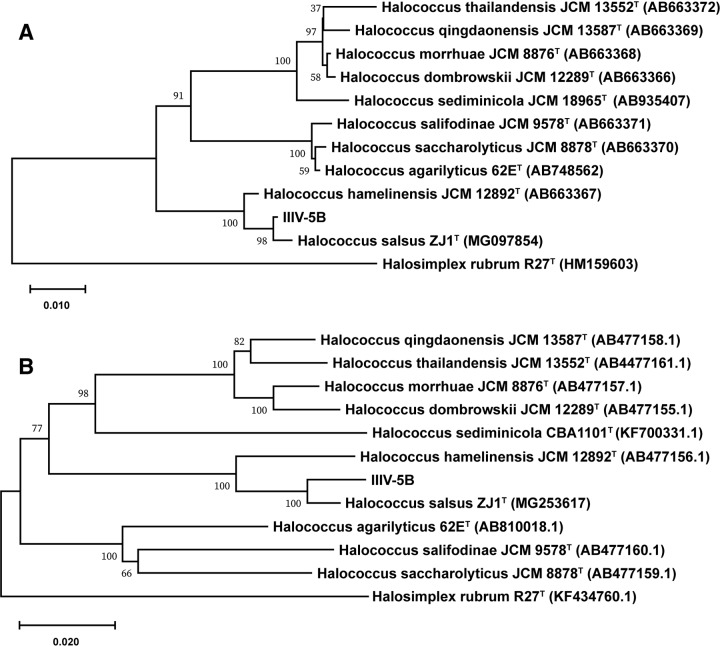
Neighbor-joining phylogenetic tree of the 16S rRNA (A) and *rpoB′* (B) gene sequences of *Halococcus* spp. and strain IIIV-5B using the p-distance model generated with MEGA X. Bootstrap values as percentages of 1,000 are shown. The bar represents 1 substitution per every 10 nucleotides. Halosimplex rubrum R27^T^ was used as the outgroup.

Sequences corresponding to the 16S rRNA and *rpoB′* genes were retrieved from the IIIV-5B annotated genome to perform taxonomical characterization. To determine the phylogenetic distance from other *Halococcus* spp., the 16S rRNA gene was obtained from the RAST server and uploaded to EZ-Taxon (17), where taxonomically close relatives were retrieved. 16S rRNA gene sequences were downloaded, aligned using ClustalW, and edited in Molecular Evolutionary Genetics Analysis X (MEGA X) software (18). The neighbor-joining tree method (19) was used to determine phylogenetic distances of 16S rRNA and *rpoB′* gene sequences between IIIV-5B and closely related strains (Fig. 1A and B). Default parameters were used for all software. The 16S rRNA gene of IIIV-5B was found to be 99.5% identical to that of Halococcus salsus ZJ1. Phylogenetic analysis of the *rpoB′* gene suggests that strain IIIV-5B might represent a new species of *Halococcus*.

### Data availability.

The draft genome sequence of strain IIIV-5B has been deposited in GenBank under the accession number QZWE00000000. The raw data have been deposited in the SRA under the accession number PRJNA490534.

## References

[1] Giri C, Ochieng E, Tieszen LL, Zhu Z, Singh A, Loveland T, Masek J, Duke N 2011 Status and distribution of mangrove forests of the world using earth observation satellite data. Glob Ecol Biogeogr 20:154–159. doi:10.1111/j.1466-8238.2010.00584.x.

[2] Kathiresan K, Bingham BL 2001 Biology of mangroves and mangrove ecosystems. Adv Mar Biol 40:81–251. doi:10.1016/S0065-2881(01)40003-4.

[3] Laanbroek HJ, Keijzer RM, Verhoeven JTA, Whigham DF 2012 The distribution of ammonia-oxidizing betaproteobacteria in stands of black mangroves (*Avicennia germinans*). Front Microbiol 3:1–11. doi:10.3389/fmicb.2012.00153. 22536201 PMC3333478

[4] Alongi DM 2008 Mangrove forests: resilience, protection from tsunamis, and responses to global climate change. Estuar Coastal Shelf Sci 76:1–13. doi:10.1016/j.ecss.2007.08.024.

[5] Twilley RW, Lugo AE, Patterson-Zucca C 1986 Litter production and turnover in basin mangrove forests in Southwest Florida. Ecology 67:670–683. doi:10.2307/1937691.

[6] López-Portillo J, Ezcurra E 1985 Litter fall of *Avicennia germinans* L. in a one-year cycle in a mudflat at the Laguna de Mecoacan, Tabasco, Mexico. Biotropica 17:186–190. doi:10.2307/2388215.

[7] McKee KL, Faulkner PL 2000 Restoration of biogeochemical function in mangrove forests. Restor Ecol 8:247–259. doi:10.1046/j.1526-100x.2000.80036.x.

[8] Soto-Ramírez N, Sánchez-Porro C, Rosas-Padilla S, Almodóvar K, Jiménez G, Machado-Rodríguez M, Zapata M, Ventosa A, Montalvo-Rodríguez R 2008 *Halobacillus mangrovi* sp. nov., a moderately halophilic bacterium isolated from the black mangrove *Avicennia germinans*. Int J Syst Evol Microbiol 58:125–130. doi:10.1099/ijs.0.65008-0. 18175696

[9] Schoop G 1935 *Halococcus litoralis*, ein obligat halophiler Farbstoffbildner. Dtsch Tierarztl Wochenschr 43:817–820.

[10] Sehgal SN, Gibbons NE 1960 Effect of some metal ions on the growth of *Halobacterium cutirubrum*. Can J Microbiol 6:165–169. doi:10.1139/m60-018. 14444586

[11] Bolger AM, Lohse M, Usadel B 2014 Trimmomatic: a flexible trimmer for Illumina sequence data. Bioinformatics 30:2114–2120. doi:10.1093/bioinformatics/btu170. 24695404 PMC4103590

[12] Bankevich A, Nurk S, Antipov D, Gurevich AA, Dvorkin M, Kulikov AS, Lesin VM, Nikolenko SI, Pham S, Prjibelski AD, Pyshkin AV, Sirotkin AV, Vyahhi N, Tesler G, Alekseyev MA, Pevzner PA 2012 SPAdes: a new genome assembly algorithm and its applications to single-cell sequencing. J Comput Biol 19:455–477. doi:10.1089/cmb.2012.0021. 22506599 PMC3342519

[13] Seemann T 2014 Prokka: rapid prokaryotic genome annotation. Bioinformatics 30:2068–2069. doi:10.1093/bioinformatics/btu153. 24642063

[14] Brettin T, Davis JJ, Disz T, Edwards RA, Gerdes S, Olsen GJ, Olson R, Overbeek R, Parrello B, Pusch GD, Shukla M, Thomason JA III, Stevens R, Vonstein V, Wattam AR, Xia F 2015 RASTtk: a modular and extensible implementation of the RAST algorithm for building custom annotation pipelines and annotating batches of genomes. Sci Rep 5:8365. doi:10.1038/srep08365. 25666585 PMC4322359

[15] Aziz RK, Bartels D, Best A, DeJongh M, Disz T, Edwards RA, Formsma K, Gerdes S, Glass EM, Kubal M, Meyer F, Olsen GJ, Olson R, Osterman AL, Overbeek RA, McNeil LK, Paarmann D, Paczian T, Parrello B, Pusch GD, Reich C, Stevens R, Vassieva O, Vonstein V, Wilke A, Zagnitko O 2008 The RAST server: Rapid Annotations using Subsystems Technology. BMC Genomics 9:75. doi:10.1186/1471-2164-9-75. 18261238 PMC2265698

[16] Overbeek R, Olson R, Pusch GD, Olsen GJ, Davis JJ, Disz T, Edwards RA, Gerdes S, Parrello B, Shukla M, Vonstein V, Wattam AR, Xia F, Stevens R 2014 The SEED and the Rapid Annotation of microbial genomes using Subsystems Technology (RAST). Nucleic Acids Res 42:D206–D214. doi:10.1093/nar/gkt1226. 24293654 PMC3965101

[17] Yoon SH, Ha SM, Kwon S, Lim J, Kim Y, Seo H, Chun J 2017 Introducing EzBioCloud: a taxonomically united database of 16S rRNA gene sequences and whole-genome assemblies. Int J Syst Evol Microbiol 67:1613–1617. doi:10.1099/ijsem.0.001755. 28005526 PMC5563544

[18] Kumar S, Stecher G, Li M, Knyaz C, Tamura K 2018 MEGA X: Molecular Evolutionary Genetics Analysis across computing platforms. Mol Biol Evol 35:1547–1549. doi:10.1093/molbev/msy096. 29722887 PMC5967553

[19] Saitou N, Nei M 1987 The neighbor-joining method: a new method for reconstructing phylogenetic trees. Mol Biol Evol 4:406–425. doi:10.1093/oxfordjournals.molbev.a040454. 3447015

